# Antiviral Activity of Binase against the Pandemic Influenza A (H1N1) Virus

**Published:** 2013

**Authors:** R. Shah Mahmud, O.N. Ilinskaya

**Affiliations:** Institute of Fundamental Medicine and Biology, Kazan (Volga Region) Federal University, Kremlyovskaya Str., 18, Kazan, Russia, 420008

**Keywords:** Bacillus intermedius ribonuclease, influenza A (H1N1) virus, A549 epithelial cell, cytotoxicity, antiviral activity

## Abstract

The lack of effective antiviral drugs restricts the control of the dangerous RNA-containing influenza A (H1N1) virus. Extracellular ribonuclease of Bacilli (binase) was shown to manifest antiviral activity during single- and multi-cycle viral replication in the range of concentrations non-toxic to epithelial cells and 0.01-0.1 multiplicity of infection. During antiviral treatment for 15-30 min, the concentration of 1 μg/ml binase reduced the amount of focus-forming units of viruses by a factor of 3-10 and suppressed the virus-induced cytopathic effect in A549 human lung cells. The possible mechanisms of interaction between the virus and enzyme are discussed. Positive charges in both binase and viral hemagglutinin cause electrostatic interaction with negatively charged sialic acid on the host cell’s surface followed by its penetration into the cell. Capsid elimination and release of viral RNA from endosome to the cytoplasm allows catalytic RNA cleavage by internalized binase. The data obtained confirm that binase is an effective antiviral agent against the pandemic influenza A (H1N1) virus. Certain progress in this field is associated with clarifying the detailed mechanism underlying the antiviral action of binase and development of the most effective way for its practical use.

## INTRODUCTION

Over the past decades, researchers have focused on ribonucleases (RN ases) as potential therapeutic agents. Some cytotoxic RN ases are cancer-selective [1-3] and antivirally active [4, 5]. These properties are inherent to RN ases of different origins; the most well-examined ones are onconases from oocytes of the northern leopard frog *Rana pipiens*, BS-RN ase from bovine testicles, and microbial RN ases from *Bacillus amyloliquefaciens* and *B. intermedius *(the new name of the species is* B. pumilus *[6]), barnase and binase, respectively. The pancreatic ribonuclease of cattle pancreas, commercially known as Ribonuclease amorphous, can be used to treat sinus infections and tick-borne encephalitis. However, an intracellular RN ase inhibitor from human cells can decrease the activity of mammal RN ases [7], thus restricting their medicinal use. In contrast to RN ase A and RN ase from human eosinophils, onconase and BS-RN ase can suppress the replication of human immunodeficiency viruses type 1 in H9 leukemia cells without a toxic effect on the infected cells [4]. Binase, when injected intramuscularly into a site of street rabies virus inoculation in mice, guinea pigs, and rabbits, has a considerable protective effect (40-67%) but does not suppress vaccine-induced antirabic immunity [5, 8]. An important point is that binase does not induce the synthesis of specific markers of the immune response, CD69 antigen and γ-interferon, in populations of CD8^+^ and CD4^+^ T lymphocytes; this fact indicates that the enzyme is devoid of the superantigenic properties inducing the polyclonal T-cell response [9]. Binase has been shown to decrease the infectious titer of the influenza type A (A/PR/8/34, A/Odessa/2882/82) and type B (B/Leningrad/369/76) viruses by two orders of magnitude, this activity being comparable with the activity of remantadin against the influenza A virus [10].

The search for effective antivirus products is an urgent task justified by the wide variability and global distribution of viruses. The aim of this study was to examine the action of binase on the pandemic influenza A/Hamburg/04/09 (H1N1) virus, the causative agent of the 2009 influenza epidemics. We found that a short-term (15-30 min) treatment of viruses with increasing concentrations of binase caused a proportional 3- to 10-fold decrease in the virus’ ability to infect A549 lung adenocarcinome cells. A binase concentration of 1 μg/mL was the most efficient and caused no inhibition of epithelial cells viability; this fact suggests that binase could be a promising antiviral agent.

## EXPERIMENTAL

**Bacterial RNase**

**Fig. 1:**
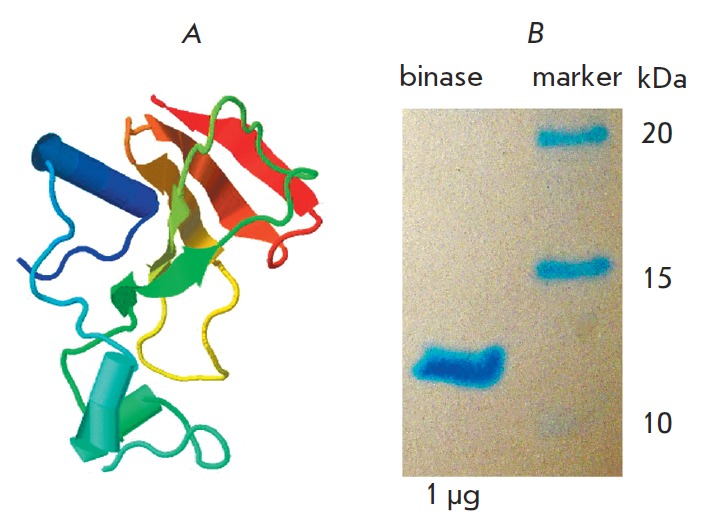
Three-dimensional structure of RNase from *Bacillus pumilus *obtained using the Jmol program (www.jmol.org; binase PDB id: 1buj) (A); electrophoregram confirming the purity of binase (B)

The guanyl-specific RN Ase from *B. pumilus *7P, binase, (12.2 kDa, 109 amino acid residues, p*I *9.5) was homogenously isolated from the culture fluid of *Escherichia coli *BL21 carrying the pGEMGX1/ent/Bi plasmid, according to A. Schulga *et al*. [11]. The molecular weight of binase, as well as its catalytic activity against synthetic substrates and high-polymeric yeast RN A, was already known (see *Fig. 1* A and [12, 13], respectively); specimen purity was confirmed by experiments (*Fig. 1* B).

**Cell cultures**

A549 (lung adenocarcinoma epithelial cell line) and MDCK II canine cocker spaniel kidney (from the collection of the Institute of Medicinal Virology Justus Liebig University, Giessen, Germany) were cultivated in DMEM supplemented with penicillin (100 U/mL), streptomycin (100 U/mL), and a 10% fetal bovine serum at 37°C and 5% CO2.

**Strain of the A/Hamburg/04/09 virus (H1N1) of influenza type A**

**Fig. 2:**
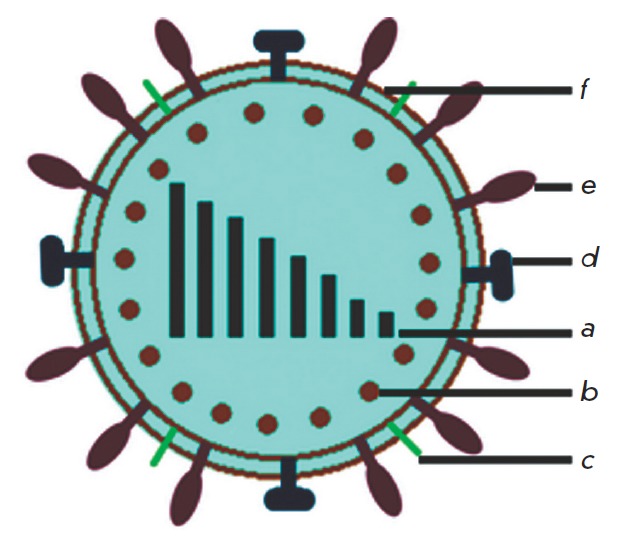
Schematic representation of the influenza A (H1N1) virus. (a) 8 molecules of viral RNA coding the PB2, PB1, PA, HA, NP, NA, M (M1, M2) and NS (NS1, NS2) proteins; (b) structural protein M1; (c) integral membrane protein of the M2 ion channel; (d) neuraminidase; (e) hemagglutinin; (f) viral lipid bilayer

The strain of the A/Hamburg/04/09 virus (H1N1) of influenza type A was obtained from the collection of the Giessen University in the form of a virus fluid. The virus material was stored at -80°C. Schematic representation of the virus and its main components is provided in *Fig. 2*.

**Cell viability**

Cell viability in the presence of binase was determined from the activity of mitochondrial dehydrogenases converting the colorless derivative of the tetrazolium dye 3-(4,5-dimethylthiazol-2-yl)-2,5-diphenyltetrazolium bromide (MTT ) (Sigma, Germany) into purple formazan crystals [14]. Staining intensity after a 24 or 48 h incubation of cells in the presence of 0.01-1000 μg/mL binase was determined from the absorbance of the formazan crystals dissolved in dimethylsulfoxide at 590 nm.

**Ribonuclease activity**

Ribonuclease activity in the culture medium of A549 cells was assessed from the amounts of acid-soluble products of the hydrolysis of yeast high-polymeric mRN A [15]. The amount of the enzyme that increased the optical density by one optical unit at 260 nm after incubation at 37°C for 1 h, calculated per mL of the enzyme solution, was taken as the activity unit.

**Number of virus particles**

The number of virus particles in the initial phage suspension was determined with a conventional hemagglutination assay of a 1.5% chicken erythrocyte suspension [16, 17]. The number of virus particles was expressed in hemagglutination units (HU) per mL, that is, the maximum dilution of virus fluid able to cause hemagglutination of erythrocytes.

**Infectious titer of the virus**

An infectious titer of the virus was determined with the immunohistochemical techniques according to the number of focus-forming units (FFU) [18]. A virus suspension was added to the MDCK II monolayer and cocultured at ambient temperature in the dark for 1 h; the virus suspension was then removed. The cells were further cultivated at 37°C and 5% CO2 in a DMEMAvicel supportive medium containing 1.25% of microcrystalline cellulose (FMC, Belgium), 0.36% of bovine serum albumin, and 1 μg/mL of trypsin treated with the TPCK chemotrypsin inhibitor (Sigma, USA). After 28 h of incubation, the culture medium was discarded, the cells were treated with ice Triton X-100 for 90 min, with mouse antibodies against the NP-protein of the influenza virus, and with the secondary anti-mouse antibodies conjugated to horseradish peroxidase (HRP) (Santa Cruz Biotechnology, USA); and stained with AEC (Sigma, USA) in dimethylformamide. Thereafter, the plate was scanned to estimates the FFU number. An infectious titer was expressed in FFU/mL of the virus fluid.

**Virus reproduction**

**Fig. 3:**
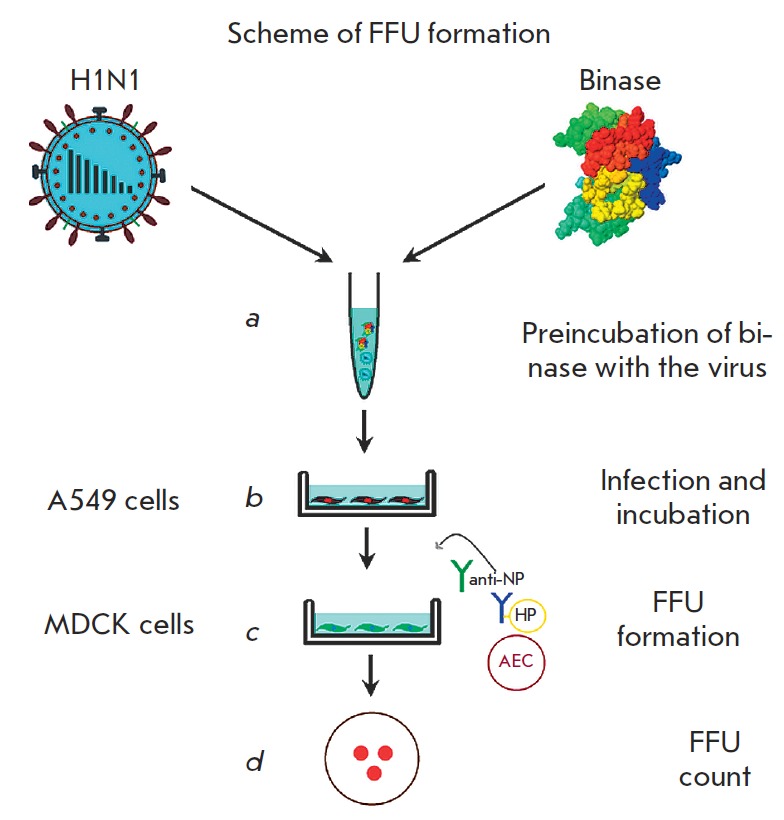
Scheme of the experiment on FFU formation. (a) preincubation of viruses with binase (15–60 min); (b) A549 cells infected with binase-treated viruses, followed by incubation for 12–24 h; (c) A549 cell culture fluid tested for the presence of viruses detected by FFU in the MDCK cell culture by adding a primary viral anti-NP antibody and horseradish peroxidase conjugated anti-mouse secondary antibody after 28 h of cultivation; (d) direct count of FFU

Virus reproduction was assayed with a 1-day-old A549 monolayer culture (3 X 104 cells per well); the infection rate was 1 or 10 virus particles per 100 cells [multiplicity of infection (MOI) 0.01 or 0.1, respectively]. The binase effect on the virus infectivity was analyzed with the infection of A549 cells with RN ase preincubated with the virus for 15-60 min. The cells were dark-incubated at room temperature for 1 h to adsorb the virus; the unadsorbed virus was removed, and the infected cells were incubated in DMEM supplemented with 0.36% of bovine serum albumin and 1 μg/mL of TPCK trypsin at 5% CO_2_ and 37°C. After incubation for 12 to 24 h (single-cycle or multi-cycle virus reproduction, respectively), the supernatant was discarded and assayed for the FFU number. The cells were washed with a phosphate buffer and stained with 1.25% Coomassie Brilliant Blue (Merck, Germany) to visualize the cells that survived the infection. The experimental scheme is shown in Fig. 3.

**Statistical analysis**

The statistical analysis of the results from four runs of each experiment was performed by standard methods in Microsoft Excel 2010 and the SigmaPlot 10 software.

## RESULTS AND DISCUSSION

**Binase cytotoxity against A549 adenocarcinoma cells**

**Fig. 4:**
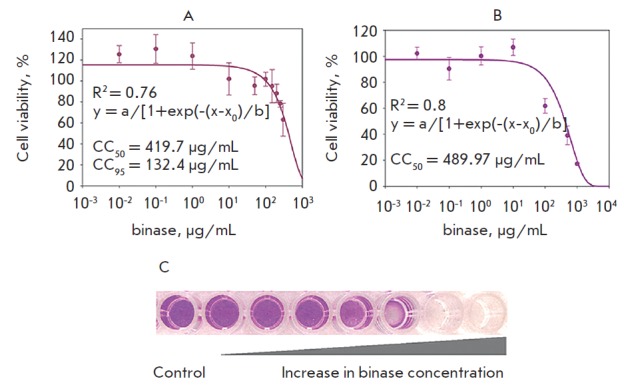
Cell viability of A549 at different binase concentrations during 24 h (A) and 48 h (B) of incubation. The R-squared value calculated by SigmaPlot 10. CC_50_ and CC_95_ – the binase concentration which induces 50% and 5% cell death, respectively. (C) Visualization of A549 cell death at increasing binase concentration (MTT assay)

Binase at a concentration approaching 1 mg/mL of the medium (corresponding to 82 μM) exerts a concentration- and time-dependent inhibiting (up to the total cell lethality) effect on the viability of A549 human lung adenocarcinoma cells. The cytotoxic binase concentration that caused 50% cell lethality (CC50) was 420-490 μg/mL after a one- or two-day exposure, respectively (Fig. 4 A, B). A concentration of 133 μg/mL caused 5% cell lethality during 24 h (CC95) (Fig. 4A). This value was significantly lower (15 μg/mL) for a 48-h treatment (the data are not presented). Thus, the binase concentrations that have no toxic effect on the cells during 1-day exposure are below 133 μg/mL. These data are consistent with the values of binase cytotoxicity against A549 cells assessed earlier with the WST assay and cytometry [19]. Since binase cytotoxicity against malignant lung epithelial cells is more pronounced than their activity against normal cells [20], one can assume that even a tenfold increase in the RN ase concentration may have no negative effect on the viability of normal epithelial cells.

Most RN ases involved in antiviral cell protection are synthesized by the host cells, and these enzymes direct the cells towards apoptotic death. In animals, the antiviral effect is exerted by ribonucleases from the RN Ase A family [21, 22], including RN ase L, whose activation causes apoptosis in the infected cells [23]. Eosinophil ribonucleases reduce the *in vitro *infectivity of virus particles by penetrating into a virus’ capsid and destroying its viral genomic RN A [24]. Examination of external RN ases showed that a pancreatic RN ase has anti-influenza activity on chicken embryos lacking a mammal ribonuclease inhibitor but had no inhibitory activity on mice [10]. Onconase can destroy the RN A of the human immunodeficiency virus while not affecting the RN A of the host cells [25]; however, a similar frog RN ase from *Rana catesbeiana *not only inhibits the replication of the Japanese encephalitis virus, but also stimulates apoptosis in virus-infected cells [26]. We found that when used at the aforementioned concentrations, binase causes no death of epithelial cells and, in addition, has no immunogenic properties and does not induce a superantigenic T-cell response [9]. This fact significantly increases the potential for the practical use of RN ase.

**Binase reduces the infectious titer of the influenza A (HIN1) virus**

**Fig. 5:**
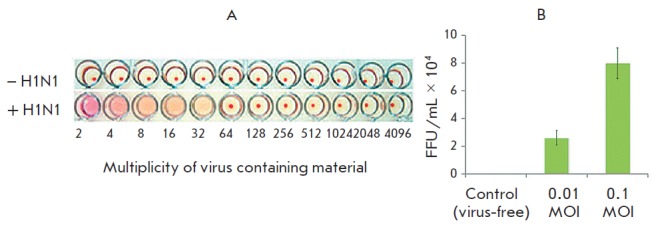
Analysis of virus-containing material for the existence of viral particles by HA unit formation calculated as a multiplicity of dilutions (A) and FFU after cultivation of A549 cells for 24 h with different MOIs (B)

The hemagglutination assay showed that the initial virus fluid contained the A/Hamburg/04/09 virus with a hemagglutination titer of 32 HA units/mL (Fig. 5A). This value indicates that the content of virus particles in the suspension is sufficient, making it possible to analyze virus resistance to antiviral agents. Exposure of A549 cells to the virus at 0.1 and 0.01 MOI showed its high infectivity, while an increase in infection multiplicity was followed by an increase in the FFU number in the plate wells (Fig. 5B). The predicted rate of the virus material infectious titer was 5.8 X 10^6^ FFU/mL.

To determine the antiviral effect of binase, the virus was co-preincubated with the enzyme at concentrations of less than cytotoxic (10^-4^-10^1^ μg/mL) for 30 or 60 min; the A549 cells were infected with the resulting suspension, and the degree of their infection at 0.1 MOI was estimated.

**Fig. 6:**
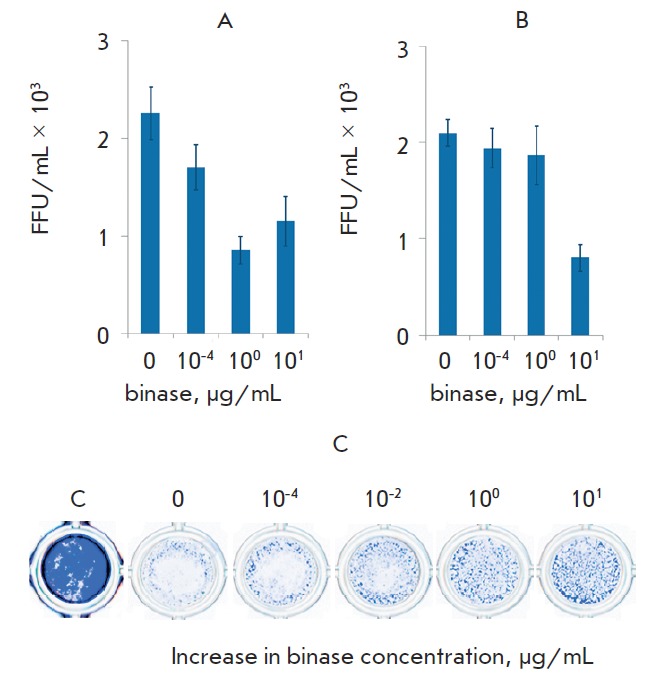
Reduction of viral infectious units under binase treatment during 30 min (A) and 60 min (B) after preincubation with the enzyme; enhancement of survival of virusinfected cells after treatment with different concentrations of binase and incubation during 12 h. Visualization of A549 cells using a methylene brilliant blue dye (C)

At a single-cycle replication of virus, when the infected cells were cultivated for 12 h, the virus titer was proportional to the increase in the binase concentration (Fig. 6). Treatment with 1 μg/mL binase reduced virus reproduction threefold (Fig. 6A).

**Fig. 7:**
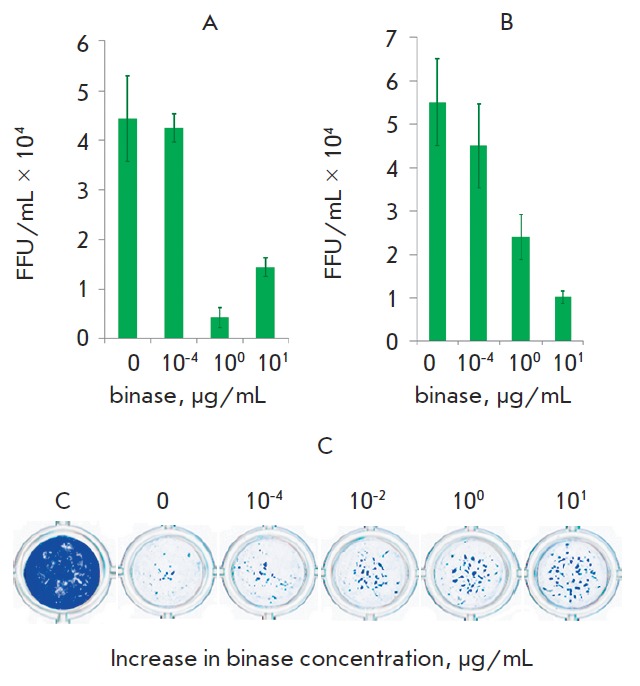
Reduction of viral infectious units under binase treatment during 30 min (A) and 60 min (B) after preincubation with the enzyme; enhancement of survival of virusinfected cells after treatment with different concentrations of binase and incubation during 24 h. Visualization of A549 cells using a methylene brilliant blue dye (C)

At a multi-cycle replication for 24 h, the antiviral effect of binase was higher: after 60 min of treatment with 10 μg/mL binase, virus reproduction decreased 6-fold (Fig. 7B). Death of A549 cells was caused by the cytopathic effect of the virus, both at single-cycle and multi-cycle replications (Figs. 6C, 7C, binase-free wells). However, the number of survived cells in a monolayer increased with the increasing binase concentration used for virus treatment. Maximum (up to 10-fold) inhibition of virus reproduction was observed one day after cell exposure to the virus preincubated with 1 μg/mL binase for 30 min (Fig. 7A).

**Fig. 8:**
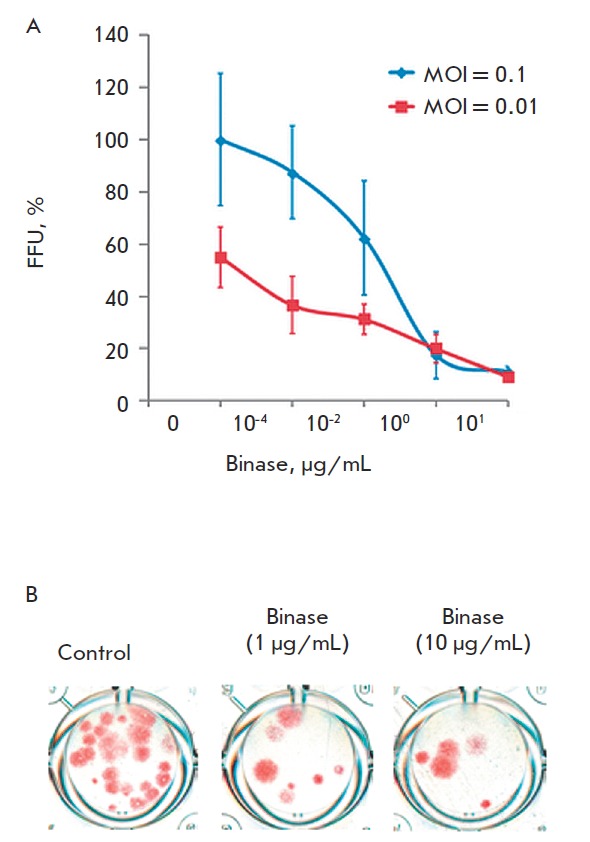
Reduction of viral infectious units under binase treatment after preincubation with the enzyme during 15 min (A) and visualization the FFU decrease in the MDCK cell culture. The value of FFU/mL of 0.1 MOI without treatment with binase was taken as 100%

The maximum effect of binase was achieved after multi-cycle virus replication; this was the reason for further assessment of the antiviral effect of binase one day after infection: the duration of the virus-enzyme preincubation period varied from 15 to 60 min, and the levels of A549 infection differed by two orders of magnitude (0.1 and 0.01 MOI) (Fig. 8A, Table 1). The antiviral effect of binase depended both on the period of co-preincubation with the virus and on the degree of virus infection. The high infection level (0.1 MOI) could provide a higher possibility of virus contacts with binase molecules and, therefore, stronger binase effect compared to a low level of cell infectivity (0.01 MOI) (Table 1).

**Table 1:** Decrease in virus infectious titer compared to that in the initial viral fluid at multi-cycle infection under the binase effect depending on the cell infectivity level and duration of virus preincubation with the enzyme

Binase, μg/mL	Preincubation of virus with binase, min					
	15	30	60	15	30	60
	0.1 MOI			0.01 MOI		
0	100 ± 25.3	100 ± 19.2	100 ± 18.1	100 ± 21.0	100 ± 7.4	100 ± 35.4
1	17.5 ± 9.0*	9.7 ± 4.5*	43.6 ± 9.4*	36.4 ± 9.8*	27.5 ± 3.5*	93.8 ± 36.9

*Significant differences with binase treatment-free control. Virus titer in the initial material without binase treatment is taken as 100%.

Independently of the cell infectivity level, the maximum antiviral effect was observed after 30 min of binase- virus co-preincubation; a longer period (60 min) of co-preincubation caused a threefold decrease in the antiviral effect (Table 1). With a low (0.01 MOI) level of cell infection, the antiviral activity of binase after 15 and 30 min enzyme treatments were virtually the same; therefore short-term (15 min) virus incubation with RN ase can be regarded as sufficient for achieving the optimal antiviral activity. During the preincubation period (15 min), 1 μg/mL of binase decreased the number of virus particles in cells approximately sixfold at 0.1 MOI and threefold at 0.01 MOI (Table 1). An increase in the RN ase concentration to 10 μg/mL caused no decrease in the virus titer after a 30min treatment (Figs. 6A, 7A) but enhanced the antiviral effectiveness of binase after 60-min preincubation (Figs. 6B, 7B).

Thus, binase concentrations not toxic for A549 cells (1-10 μg/mL) inhibit the reproduction of the influenza A (H1N1) virus when pretreated with binase for 15-30 min.

**Table 2:** Increase in catalytic activity of binase in culture medium of A549 cells (U/mL) after 48 h cultivation

Binase, μg/mL	0 h	48 h
0	5.3 ± 1.4	63.0 ± 9.3
1	7107.1 ± 770.7	4078.3 ± 462.7
10	64600.0 ± 6648.7	45000.0 ± 5870.0

Immunofluorescent methods demonstrated that binase had penetrated into the A549 cells even within the first hours of incubation [28]. The catalytic activity of the enzyme is known to be maintained in myeloid progenitor cells for 48 h [28]. We registered a decrease in binase catalytic activity in a culture medium of A549 cells treated with 1 and 10 μg/mL binase (Table 2); this fact also attests to enzyme penetration into a cell. The mechanism of ribonuclease internalization is conditioned by the interaction between the cationic protein and the negative charge of the tumor cell surface; further cell penetration is provided by endocytosis [1]. Since the virus penetrates into a cell in a similar way, binase-virus interaction can take place inside the cell as well (in particular, inside endosomes). The receptors of the influenza virus hemagglutinin on the host cell surface carry a negative charge provided by the sialic acid [29, 30]; hence, binase can interact with the surface of such cells via the electrostatic mechanism and penetrate into them independently of the virus. In the virus-infected cells, binase will cleave the viral RN A for at least 48 h until it is hydrolyzed by cell proteases [28]. It is noteworthy that the high temperature resistance of binase and maintenance of its activity in a wide pH range [31] is one of the significant factors conditioning its use.

Binase demonstrates antiviral activity against the viruses of rabies, the hoof-and-mouth disease, some plant viruses, and the seasonal influenza virus [8, 10, 32]. Up to 500 thousand human deaths are caused annually by the influenza virus. According to the WHO, the pandemic influenza caused by the A virus (H1N1) alone affected 414,000 humans, 5,000 cases ending in a fatal outcome (www.who.int). The continuous evolution of the virus due to the antigen drift and mixing of viral genetic material limits the efficiency of currently recognized protective strategies, including vaccination and administration of neuraminidase inhibitors. This fact explains the importance of a new therapeutic strategy, whose effectiveness would be independent of a virus’ subtype. Our data show the high promise of developing the bacterial RN ase-based strategy. A binase possessing a number of advantages as compared to its eukaryotic analogs (resistance to an inhibitor of mammalian RN ases and easy production) can become the next-generation antiviral agent.

## CONCLUSION

The high death rates that accompany influenza impose a serious social and economic burden on society; therefore, containing pandemics of influenza A type/H1N1 viruses is one of the urgent tasks facing the scientific community. Rapid spreading of a viral infection can be checked by using broad-specificity drugs whose effectiveness is independent of particular mutations in the virus’ genome. We have shown that concentrations of secretable ribonuclease from *B. intermedius *(binase) e antiviral toxicity have no toxic effect on human epithelial cells. The pretreatment of viral particles with binase at approximately a 1 μg/mL concentration caused a significant (up to tenfold) decrease in virus infectivity and suppressed the development of the virus-induced cytopathic effect in a A549 human lung cell line at various multiplicity of infections both during single-cycle and multi-cycle virus reproduction. Fine-tuned mechanisms of antiviral activity need further examination, although it is conceivable that they include both the charge-charge interaction between cation binase and the negatively charged hemagglutinin receptors on the surface of a host cell and catalytic cleavage of the viral RN A inside cells. The data confirm the applicability of binase as an effective antiviral agent against the pandemic influenza A (H1N1).

## Abbreviations

FFU: focus-forming units
HA: hemagglutination
MOI: multiplicity of infection
AEC: 3-amino-9-ethylcarbazole
TPCK: L-1-Tosylamide-2-phenylethyl chloromethyl ketone
